# Evaluation of real-world referential and probabilistic patient matching to advance patient identification strategy

**DOI:** 10.1093/jamia/ocac068

**Published:** 2022-05-14

**Authors:** Shaun J Grannis, Jennifer L Williams, Suranga Kasthuri, Molly Murray, Huiping Xu

**Affiliations:** Department of Family Medicine, Indiana University School of Medicine, Indianapolis, Indiana, USA; Regenstrief Institute, Center for Biomedical Informatics, Indianapolis, Indiana, USA; Regenstrief Institute, Center for Biomedical Informatics, Indianapolis, Indiana, USA; Regenstrief Institute, Center for Biomedical Informatics, Indianapolis, Indiana, USA; Department of Pediatrics, Indiana University School of Medicine, Indianapolis, Indiana, USA; Pew Charitable Trust, Baltimore, Maryland, USA; Department of Biostatistics, IU Richard M. Fairbanks School of Public Health, Indianapolis, Indiana, USA

**Keywords:** record linkage, patient matching, patient identification, health IT policy, identity management

## Abstract

**Objective:**

This study sought both to support evidence-based patient identity policy development by illustrating an approach for formally evaluating operational matching methods, and also to characterize the performance of both referential and probabilistic patient matching algorithms using real-world demographic data.

**Materials and Methods:**

We assessed matching accuracy for referential and probabilistic matching algorithms using a manually reviewed 30 000 record gold standard reference dataset derived from a large health information exchange containing over 47 million patient registrations. We applied referential and probabilistic algorithms to this dataset and compared the outputs to the gold standard. We computed performance metrics including sensitivity (recall), positive predictive value (precision), and F-score for each algorithm.

**Results:**

The probabilistic algorithm exhibited sensitivity, positive predictive value (PPV), and F-score of .6366, 0.9995, and 0.7778, respectively. The referential algorithm exhibited corresponding sensitivity, PPV, and F-score values of 0.9351, 0.9996, and 0.9663, respectively. Treating discordant and limited-data records as nonmatches increased referential match sensitivity to 0.9578. Compared to the more traditional probabilistic approach, referential matching exhibits greater accuracy.

**Conclusions:**

Referential patient matching, an increasingly popular method among health IT vendors, demonstrated notably greater accuracy than a more traditional probabilistic approach without the adaptation of the algorithm to the data that the traditional probabilistic approach usually requires. Health IT policymakers, including the Office of the National Coordinator for Health Information Technology (ONC), should explore strategies to expand the evidence base for real-world matching system performance, given the need for an evidence-based patient identity strategy.

## INTRODUCTION

Accurate patient matching is essential to quality care. Attributing clinical records to the correct patient, or patient matching, is necessary to ensure safe, high-quality, and cost-effective patient care. Yet, patient data remain fragmented across many systems without consistent approaches to identifying patient records within and among organizations.^1^^,^^2^ Poor patient matching hinders aggregating and exchanging patient information and fragmented patient data limits care coordination, causes waste such as duplicate testing and is detrimental to patient safety.^3–6^

Improved patient identity is needed to lower care costs. Current identity management approaches result in repeated procedures, lost revenues from rejected insurance claims, and higher data management cost. A study of the Children’s Medical Center of Dallas’ matching system revealed duplicate records cost an average of nearly $100 per duplicate record and over $1100 for duplicate records associated with repeated tests or treatment delays.^7^ A 2018 Black Book Survey found that duplicate patient records cost an average of $1950 per patient per inpatient stay and over $800 per emergency department visit.^8^ According to the same study, one-third of rejected insurance claims are attributed to inaccurate patient identification, costing the average hospital $1.5 million and the US healthcare system $6 billion annually.

Patient matching approaches abound as the United States has no consistent approach to patient identity management. While recognizing the need for improved patient identity is growing^9^^,^^10^ the United States has neither a national unique patient identifier nor other uniform approaches for managing patient identities. Instead, organizations commonly identify patients using algorithms to sort through demographic records to make matches.^11^ This is due in part to the longstanding congressional prohibition of funding for establishing a unique patient identifier. In recent consideration of the issue, Congress charged HHS to recommend improvements to patient identification.^12^ However, the Senate rejected a repeal of the ban on funding to develop a unique patient identifier, maintaining the status quo.^13^

Traditional matching uses limited data, but emerging approaches may improve match accuracy. Matches are conventionally determined by comparing 2 patient demographic records field-by-field to assess similarity. Referential matching, an increasingly popular approach, instead uses large collections of demographic records, such as information from credit reporting agencies or address change records, providing a multi-record benchmark to match identities. The Pew Charitable Trusts defines referential matching as “leverag[ing] data from different sources to build a more complete profile of each patient that includes past addresses, common name spellings for individuals, and other demographic data that changes over time.”^14^ Additional data may improve accuracy, for example, when evaluating patient records that lack key identifying fields, have out-of-date data, or belong to different people who happen to share important demographic data points (such as family members). Data quality varies between health and referential sources. Therefore, referential algorithms deploy additional matching logic to accommodate these differences. To our knowledge, no studies have evaluated referential matching despite the growing adoption of these approaches and calls to study the technique.^15^^,^^16^

We do not know the accuracy of patient matching approaches. The relative performance of matching algorithms used in the US healthcare system and the quality of demographic data used for matching is largely unknown because few *in* *vivo* matching approaches are rigorously evaluated. In addition, real-world match performance may suffer due to population data characteristics that deviate from original design assumptions. For example, matching newborns is notoriously challenging due to limited identifiers, and techniques for matching adults perform poorly for this group.^16–18^ Benchmarking matching accuracy is essential for informing policy to improve patient safety, provide high-quality care, and reduce costs.

Formal evaluation of patient matching approaches is needed to improve methods and inform policy. This study aimed to evaluate the accuracy of an exemplar referential matching algorithm using real-world datasets from one of the nation’s largest health information exchanges (HIEs). This will be the first accuracy evaluation of its type. Findings can inform policymakers and delivery system decision-makers regarding the value of standardizing accuracy and benchmarking one approach, referential matching.

## MATERIALS AND METHODS

### Materials

#### Patient demographic data

We used patient records extracted from the Indiana Network for Patient Care (INPC),^19^ an HIE containing over 47 million registration records across more than 100 clinical data sources. The INPC represents a unique *in* *vivo* laboratory to evaluate patient matching methods,^20^^,^^21^ and the demographics of the INPC catchment area closely mirror the demographics of the US overall, supporting the generalizability of findings. By design, the INPC stores a distinct identity for each person as recorded by each health institution. For example, the INPC will store 2 distinct registration records for a patient with clinical encounters at hospital A and clinic B.^22^ Fields used for linkage include social security number (SSN), name (last, first, middle initial), gender, birthdate, phone number, street, and ZIP code.

### Methods

#### Linkage algorithms

We evaluate 2 linkage algorithms, probabilistic and referential. The probabilistic software implements a weighted similarity^23^ algorithm. It incorporates the following steps typical of contemporary matching algorithms. Data normalization ensures comparisons use consistent values, and includes address parsing and data standardization (eg, converting a gender of “Male” to “M”, or street line value of “AVENUE” to “AVE”). Invalid values such as names including “BABY BOY” and “TEST” or SSNs with all 9’s are excluded.

To identify the most likely matches while maintaining acceptable performance, blocking schemes gather candidates sharing at least a portion of matching fields and exclude record pairs with insufficient similarity.^24^ Blocking schemes comprise multiple combinations of features among name, birthdate, SSN, address, and phone number.

Candidate matches are scored attribute-by-attribute using a weighted similarity based on discriminating power, summed across matching attributes. For example, birthdate weight is greater than gender. Attribute similarity can be detected for each attribute and informs scoring.^25^ For example, 2 names sharing common nicknames contribute a larger weight than 2 names differing by typographical errors. Examples of match similarity types include nickname, phonetic matching for names, and character transpositions for any string of data. Standard attributes used in scoring are SSN, name, gender, birthdate, phone number, and address.

The set of attribute data is evaluated using heuristic rules for specific conditions that increase or decrease the likelihood of the match and adjust the weighted score accordingly. These conditions include familial relationships such as twins or parent/child. The records are declared a match if the final match score exceeds a configurable threshold.

The referential algorithm, in addition to probabilistic matching techniques described above, uses a curated reference dataset of all US adults and additional logic adapted to the data characteristics that vary by combination of patient and reference data. Reference data derive from commercially available, nonhealthcare sources, including credit header data and federal, state, and local government person records.

Patient demographic records are compared to reference data using a probabilistic matching approach. When a candidate referential identity is found, corresponding reference data and logic are incorporated into subsequent steps to prevent missed matches and false positives. For example, if a recently married person changed their last name and moved, historical reference data values for both last names and addresses may identify more potential candidates and improve matching outcomes, thus preventing false negatives. Logic enabled by additional reference data may uncover a candidate identity representing multiple distinct persons. For example, family members may share similar names, and referential data can further differentiate individuals, preventing false positives. To mitigate erroneous transitive linking, the referential matching logic differentiates the record type being compared (person versus reference record). While transitive linking is often desirable among source person records, links between reference data records can indicate a potential false positive scenario. Finally, source patient records that do not align to reference data may still match probabilistically to other source patient records, independent of whether the other records matched to referential data.

#### Algorithm evaluation

**Figure 1.**
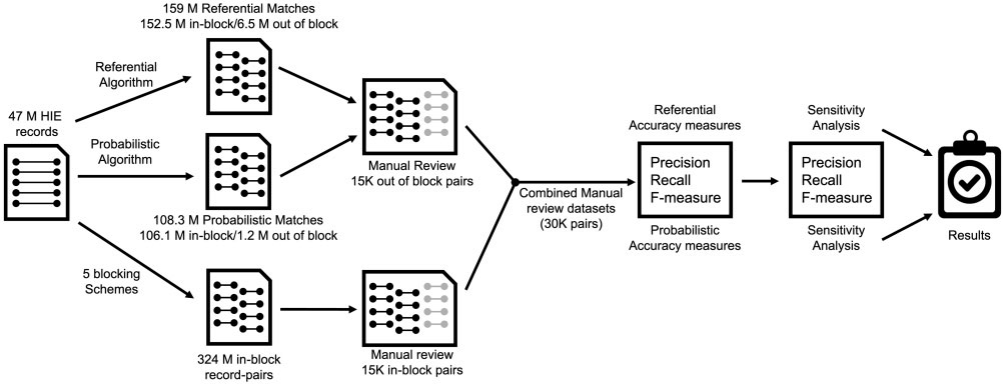
Overview of match performance evaluation. Forty-seven million HIE registration records were used to create 324 million record pairs using 5 blocking combinations. The 5 blocking schemes were: SSN, FN+TEL, DB+MB+YB+ZIP, FN-LN-YB, and DB+MB+YB+ZIP. Blocking schemes produced 53, 41.7, 133.5, 193.9, and 191.2 M record-pairs, respectively. (FN: first name, LN: last name, TEL: phone number, ZIP: zip code, MB, DB, and YB: birth month, day, and year, respectively.) The 47 million records were also evaluated by both referential and probabilistic algorithms to identify matches. In- and out-of-block record pair samples were reviewed to establish a combined reference dataset. Based on this dataset match performance metrics were calculated. A sensitivity analysis was conducted to assess match performance under more conservative matching rules.

**Figure 2.**
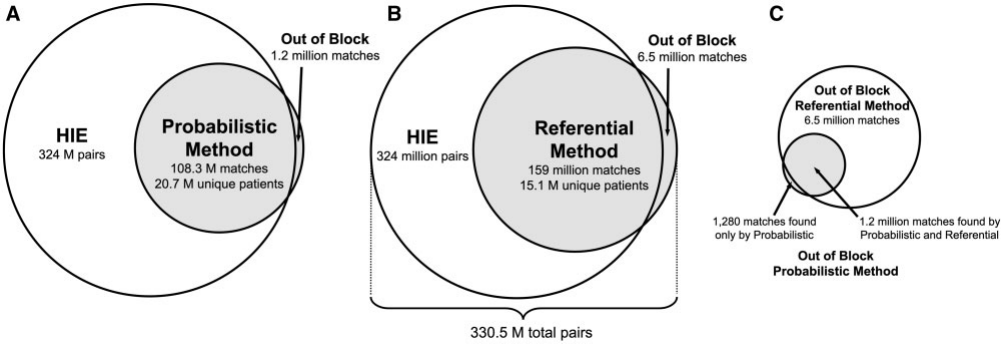
Comparison of referential and probabilistic matches relative to HIE candidate pairs formed by 5 blocking schemes. (A) Probabilistic matches. Comparison of probabilistic matches relative to HIE pairs. The probabilistic method identified 1.2 M matches outside of the HIE pairs formed by 5 blocking schemes. (B) Referential matches. Comparison of referential matches relative to HIE pairs. The referential method identified 6.5 M matches outside of the HIE pairs formed by 5 blocking schemes. Fifteen thousand pairs were randomly sampled from the 324 million in-block pairs for match analysis. An additional 15 000 pairs were sampled from the 6.5 million out-of-block matches. (C) Out-of-block probabilistic and referential matches. Comparison of out-of-block matches for probabilistic and referential methods. A total of 1280 probabilistic matches were identified by the probabilistic method only, and referential method identified 5.3 M more out–of-block matches than probabilistic.

Figure 1 summarizes the match performance evaluation. We created a set of candidate record pairs using blocking schemes to assess the linkage algorithms. Although probabilistic linkage algorithms often use blocking to reduce the potential number of record pairs in comparison, we selected these blocking schemes independent of the probabilistic linkage algorithm under evaluation since algorithm evaluation was performed separately from algorithm development. The 5 blocking schemes included validated values for SSN, first name + telephone number, last name + birthdate, date of birth + ZIP code, and last name + first name + birth year. The union of pairs formed by all blocking schemes totaled approximately 324 million record pairs. Probabilistic and referential methods identified approximately 6.5 million additional record pairs outside the blocking schemes (Figure 2), of which 93% were classified as matches by the probabilistic or referential linkage algorithms. The remaining 7% agreed on name and birthdate only, which were not captured in the 5 blocking schemes formed using preprocessed blocking field values. We calculated performance metrics using the combined within and outside of block sets, totaling 330.5 million record pairs.

*Manual* *review*. We randomly sampled 15 000 record pairs for manual review from each set (324 million record pairs within the blocking schemes and 6.5 million record pairs outside the blocking schemes). We used simple random sampling to select record pairs within the blocking schemes. We used a stratified random sample design for pairs outside the blocking schemes where strata were determined by algorithm match status (match or nonmatch) and agreement status for standardized name and birthdate. We oversampled the stratum that only agreed on standardized name and birthdate to better evaluate potential false negative matches.

Record pairs were manually reviewed to determine a gold standard match status, either match or nonmatch. For each pair, reference data for both records were included in the manual review process to aid in adjudicating match status. We used a balanced incomplete block design to assign reviewers, where 2 reviewers reviewed each pair. A third reviewer adjudicated pairs with discordant match status.

*Matching* *performance* *metrics*. We evaluated matching performance using sensitivity, positive predictive value (PPV), and F-score. The F-score, the harmonic mean of sensitivity and PPV, provides an overall measure of the matching accuracy. We estimated these metrics by comparing the results of the probabilistic and referential linkage algorithms to the manually reviewed reference. The sampling design, including strata and probability weighting, was properly accounted for when estimating and comparing the matching performance metrics.

*Sensitivity* *analysis.* Manual review is not without error, particularly when records contain sparse information. Consequently, we performed a sensitivity analysis to estimate matching performance metrics by treating discordant and limited-data records as nonmatches. For this analysis, we considered as nonmatches those record pairs that required third-reviewer adjudication due to the discrepancy between the 2 original reviews, and whose manual review and linkage algorithm match status disagreed. Further, because the referential algorithm classifies many record pairs outside of the blocking schemes that agree only on name and birthdate as nonmatch, we treated these as nonmatches to better understand linkage performance under these assumptions.

## RESULTS

### Linkage algorithms

The probabilistic algorithm identified 20.7 million unique patients among the 47 million HIE records. Of these, 13.7 million had a single demographic record. The records per patient metric for the remaining 7 million patients ranged from 2 to 179, with an average of 4.8 and a median of 4. Altogether, the probabilistic algorithm declared 108.3 million matched record pairs, of which 107.1 million were within the 5 blocking schemes, with the remaining 1.2 million outside the blocking schemes (Figure 2).

The referential algorithm identified 15.1 million unique patients, with 7.7 million having a single record. For the remaining 7.4 million with multiple records, records per patient ranged from 2 to 251, with an average of 5.4 and a median of 4. The referential algorithm declared a total of 159 million matched record pairs. Of these, 152.5 million pairs were within the 5 blocking schemes, with the remaining 6.5 million pairs.

### Matching performance

Among the 15 000 reference record pairs within the blocking schemes, 7647 (51%) were declared matches, and the remaining 7353 (49%) nonmatches. Among the 15 000 reference record pairs outside the blocking schemes, 11 187 (75%) were matched, and the remaining 3813 (25%) nonmatches. For record pairs within the blocking scheme, reviewer match status agreed for 14 412 (96.1%) pairs, while reviewer match status disagreed for 588 (3.9%) pairs, requiring adjudication by a third reviewer to establish match status. For record pairs outside the blocking scheme, reviewer match status agreed for 13 027 (86.8%) pairs, while 1973 (13.2%) required adjudication by a third reviewer. Table 1 shows the probabilistic and referential linkage results for these record pairs. The referential algorithm identified substantially more gold standard matches than probabilistic.

**Table 1.** Results from manual review of potentially matched records

Probabilistic	Referential	Total frequency	Manual review result	
			Nonmatch	Match
Within the blocking schemes				
Nonmatch	Nonmatch	7855	7351	504
Nonmatch	Match	2134	0	2134
Match	Nonmatch	2	0	2
Match	Match	5009	2	5007
Outside of the blocking schemes				
Nonmatch	Nonmatch	5972	3188	2784
Nonmatch	Match	6401	11	6390
Match	Nonmatch	1280	600	680
Match	Match	1347	14	1323

The greater number of gold standard referential matches produced improved matching performance (Table 2). Using all manually reviewed record pairs within and outside the blocking schemes, sensitivity for the probabilistic algorithm was 63.66% (95% CI: 62.64%–64.68%). Sensitivity for the referential algorithm was 93.51% (95% CI: 92.97%–94.04%), an improvement of almost 30% (95% CI: 28.88%–30.82%, *P* < 0.001). The improved sensitivity was not associated with reduced PPV. The referential algorithm exhibited a PPV of 99.96%, slightly greater than 99.95% observed for the probabilistic algorithm. With improved sensitivity and comparable PPV, the referential algorithm produced an F-score of 96.63% (95% CI: 96.34%–96.91%), a nearly 20% (95% CI: 18.14%–19.56%, *P* < 0.001) improvement in the F-score over the probabilistic algorithm.

**Table 2.** Estimated matching performance metrics based on manual review data within and outside the blocking schemes

	Probabilistic	Referential	Difference	*P*-value
Sensitivity	0.6366 (0.6264, 0.6468)	0.9351 (0.9297, 0.9404)	0.2985 (0.2888, 0.3082)	<0.001
PPV	0.9995 (0.9990, 1.0000)	0.9996 (0.9993, 1.0000)	0.0001 (−.0001, 0.0003)	0.34
F-score	0.7778 (0.7702, 0.7855)	0.9663 (0.9634, 0.9691)	0.1885 (0.1814, 0.1956)	<0.001

### Sensitivity analysis

Among manually reviewed record pairs within the 5 blocking schemes, the major discrepancy between the referential algorithm and the gold standard derived from 506 record pairs classified as nonmatches by the referential linkage algorithm but declared to be matched by manual review. Of these, 178 pairs had discordant results from the 2 reviewers and were deemed matches by the adjudicator. In the sensitivity analysis, we consider these 178 pairs as nonmatches. In addition, 5972 record pairs among the 15 000 manually reviewed record pairs outside of the blocking schemes agreed on only standardized name, gender, and birthdate. All were declared nonmatches by the referential algorithm, while manual review declared 2784 of them to be matches. We considered these 2784 pairs as nonmatches for the sensitivity analysis.

Matching performance using the sensitivity analysis assumptions is shown in Table 3. Under the assumptions in the sensitivity analysis, the referential algorithm yields a 2% greater sensitivity with the same PPV. This results in a 1.2% higher F-score, reflecting an increased concordance between the referential algorithm and the gold standard under these assumptions.

**Table 3.** Estimated matching performance metrics in the sensitivity analysis

	Probabilistic	Referential	Difference	*P* value
Sensitivity	0.6519 (0.6417, 0.6622)	0.9578 (0.9533, 0.9623)	0.3059 (0.2960, 0.3157)	<0.001
PPV	0.9993 (0.9987, 1.0000)	0.9996 (0.9993, 1.0000)	0.0003 (−0.0001, 0.0007)	0.19
F-score	0.7891 (0.7816, 0.7966)	0.9783 (0.9759, 0.9806)	0.1892 (0.1820, 0.1963)	<0.001

Sensitivity for the referential algorithm is 95.78% (95% CI: 95.33%–96.23%), which is again substantially higher than 65.19% (95% CI: 64.17%–66.23%) for the probabilistic algorithm. The PPV for the referential algorithm is slightly higher than the probabilistic algorithm. This produced an F-score of 97.83% (95% CI: 97.59%–98.06%) for the referential algorithm, superior to the 78.91% (95% CI: 78.16%–79.66%) F-score for the probabilistic algorithm.

## DISCUSSION

This study represents one of the first of its kind: an evaluation of a broadly implemented patient matching algorithm using documented methods and real-world gold standard data. We are unaware of prior instances of a commercial matching system undergoing a formal, transparent, third-party analysis using real-world demographic data. Such analyses help to establish baseline performance expectations and identify opportunities for improving match accuracy.

As the United States continues to advance a national identity strategy for healthcare, a more consistent and broadly deployed approach to objectively evaluating matching algorithms is necessary to provide transparency and support healthcare organizations in adopting evidence-based best practice guidelines for patient matching algorithms. Consequently, health IT policymakers, including the Office of the National Coordinator for Health Information Technology (ONC), should explore strategies for expanding the evidence base for real-world matching system performance and encourage the development of more consistent and transparent approaches to assessing and disseminating matching system performance.

Record pairs with referential match and probabilistic nonmatch statuses contained various combinations of changing name, address, phone number, or missing values. These reflect cases where patients appeared to change location, use both nicknames and given names, change or use multiple phone numbers, have typographical or recording errors in any of these values, or have incomplete data. Referential sources more completely captured these changing combinations of demographics over time to identify undetected relations between seemingly distinct identities, which significantly increased match sensitivity.

While 1280 record pairs were labeled probabilistic match and referential nonmatch, it is important to recall that pairs derived from the out-of-block set represented only 4% of all declared matches and had a limited effect on overall match performance. With several missing or mis-recorded values, manual reviewers classified 47% of these cases as nonmatches. Referential logic either differentiated distinct but similar appearing clinical identities or lacked sufficient data to declare a match or nonmatch.

Referential matching exhibited superior sensitivity compared to probabilistic while preserving a high PPV, suggesting that improved accuracy associated with referential matching results from additional demographic data and expanded matching logic made possible by the additional data.

On the surface, these results may seem to compare less favorably to matching systems claiming to achieve 99% or greater accuracy.^26^ However, these comparisons can be misleading for multiple reasons. First, methods and measures for evaluating real-world operational matching performance are often underspecified or undefined and rarely undergo peer-review analysis, making it difficult to compare these methods. Second, the quoted performance of many matching systems reflects the success of the algorithm assisted by human adjustment, not the system alone. Note that no matching system adjustments were made for this analysis. We note that accuracy measurements are influenced by prior assumptions, highlighted by discrepancies we observed related to records with less data: among records agreeing only on name, gender, and birthdate, we more often declared matches than the probabilistic and referential match algorithms based on our team’s nearly 20-year experience verifying HIE records for matching research.^19^^,^^25^^,^^27–29^

### Limitations

This study has 2 main limitations. First, reference data quality, including data completeness and accuracy, influences referential matching performance. Thus, referential matching systems using reference data with differing coverage for population subgroups such as children and the homeless or reference data with error rates varying from the system evaluated may yield different results. Second, while the data included in this study represent a broad spectrum of healthcare settings, which supports the likelihood for generalizability, our analysis used data specific to Indiana health systems. Consequently, results may vary in environments with markedly differing demographic data characteristics.

## CONCLUSION

Referential patient matching demonstrated notably greater accuracy than a more traditional probabilistic approach without typically required adaptation of the algorithm to the data. This information is important because independently and formally quantifying patient matching accuracy is essential for effective policy development and improved patient safety. Pragmatically, the findings from this study provide transparency and can be meaningful to those making decisions regarding identity matching solutions. Consequently, health IT policymakers, including ONC, should explore strategies to expand the evidence base for real-world matching performance.

## FUNDING

Study funding was provided by the California HealthCare Foundation, who holds a minority stake in the referential matching software vendor Verato.

## AUTHORS CONTRIBUTIONS

SJG participated in the conception and design of the research, analysis, and interpretation of data, and drafted and revised the content. JLW substantially contributed to the acquisition, analysis, and interpretation of data for the research, and reviewed and revised the work critically. SK contributed to the design of the study, supported acquisition and analysis of data, and revised and critically reviewed manuscript drafts. MM contributed to the conception of the study, and critically reviewed and revised the manuscript. HX participated in the conception and design of the research, analysis, and interpretation of data, and drafted and revised the content.

## CONFLICT OF INTEREST STATEMENT

None declared.

## DATA AVAILABILITY

Due to privacy and ethical concerns related to protected health information, the data cannot be made available.
